# Efficiency of a Novel Multifunctional Corrosion Inhibitor Against Biofilms Developed on Carbon Steel

**DOI:** 10.3389/fbioe.2022.803559

**Published:** 2022-01-21

**Authors:** Benjamin Tuck, Nadia Leinecker, Elizabeth Watkin, Anthony Somers, Maria Forsyth, Laura L. Machuca

**Affiliations:** ^1^ Curtin Corrosion Centre, WA School of Mines: Minerals, Energy and Chemical Engineering, Curtin University, Bentley, WA, Australia; ^2^ Curtin Medical School, Curtin University, Bentley, WA, Australia; ^3^ Institute for Frontier Materials, Deakin University, Burwood, VIC, Australia

**Keywords:** biofilm, biofouling, corrosion inhibitor, microbiologically influenced, biocide

## Abstract

In natural environments, populations of microorganisms rapidly colonise surfaces forming biofilms. These sessile communities comprise a variety of species which contribute to biofouling and microbiologically influenced corrosion (MIC), especially on metals. Species heterogeneity in natural systems confers higher tolerance to adverse conditions such as biocide treatment compared with single species laboratory simulations. Effective chemical treatments to combat recalcitrant biofilms are often dangerous to apply; both to operators and the environment, and face international embargoes. Today, there is a drive to exchange current toxic and environmentally hazardous biocides with less harmful compounds. One effective method of achieving this goal is to generate multi-functional compounds capable of tackling corrosion and biofilm formation simultaneously, thus reducing the number of compounds in dosing procedures. In a previous study, a novel corrosion inhibitor demonstrated biocidal effects against three marine isolates during the early stages of biofilm formation. The compound; CTA-4OHcinn, holds great promise as a multi-functional inhibitor, however its effect on complex, multi-species biofilms remains unknown. Here we evaluate CTA-4OHcinn biocidal capacity against multi-species biofilms developed from oilfield samples. Mature biofilms were developed and treated with 10 mM CTA-4OHcinn for 4 h. The effects of the compound were assessed using mean probable number (MPN), adenosine triphosphate (ATP) quantification, scanning electron microscopy (SEM) and confocal laser scanning microscopy (CLSM). Results demonstrate that CTA-4OHcinn significantly reduces the viability of mature biofilms, supporting previous demonstrations on the secondary function of CTA-4OHcinn as a biocide. CLSM results further indicate that CTA-4OHcinn targets the cell membrane resulting in lysis. This finding complements the established corrosion inhibition function of CTA-4OHcinn, indicating the compound is a true multi-functional organic corrosion inhibitor.

## 1 Introduction

Microorganisms are ubiquitous in the marine environment as free-floating planktonic cells (Ista et al., 2004; Van Tol et al., 2017). Planktonic microorganisms contact submersed surfaces, where they attach and form diverse communities surrounded by extracellular polymeric substances (EPS) (Kumar et al., 2011; El-Kirat-Chatel et al., 2017). EPS typically consists of nucleic acids, polysaccharides, proteins and other organic and inorganic substances (Saravanan and Jayachandran, 2008; Flemming and Wingender, 2010; Kavita et al., 2014). This biofilm phenotype is the preferred lifestyle for most bacteria, owing to the numerous benefits afforded by the community and the EPS (Kumar et al., 2011). For example, the EPS can provide protection from desiccation, chemical treatment, and mechanical stress (Dieltjens et al., 2020). Among other benefits, the biofilm lifestyle also provides a source of nutrition and a reservoir for antimicrobial or biocide tolerance (Schlafer and Meyer, 2017). The mature biofilm population with EPS can be as much as 1,000 times more tolerant to chemical treatments compared to the free-floating planktonic phenotype (Dieltjens et al., 2020). Unlike planktonic cells, metabolic functions of mature biofilms are known to impact the base substrate resulting in serious economic, environmental and health impacts.

In the marine environment, a plethora of recent studies have investigated the attachment and biofilm formation of bacteria to engineered systems, since progression of the biofilm results in biofouling and microbiologically influenced corrosion (MIC) (Tuck et al., 2020; Albahri et al., 2021; Catubig et al., 2021; Qian et al., 2021; Tran et al., 2021). MIC is particularly costly, expected to contribute around 20–50% of the total $4T USD global corrosion costs (Little and Lee, 2007; Li et al., 2018). The initial biofilm stages involving attachment and adhesion are considered critical to biofouling and MIC; therefore attracting considerable attention in recent years (Javed et al., 2015; Javed et al., 2016; Caruso, 2020). If biofilm formation can be limited at these early stages, deleterious impacts of biofilm formation may be evaded. Although research from the last two decades has greatly enhanced the scientific understanding of initial biofilm formation, no natural or artificial surface can totally prevent bacterial attachment (Vanithakumari et al., 2018; Tuck et al., 2020). In reality, a mature biofilm will develop on any substrate exposed to the marine environment if effective treatments are not regularly applied.

Biocides are a primary tool for controlling bacterial attachment and mature biofilms in marine environments. As biofilms develop tolerance to chemical treatment, the use of potent and potentially harmful biocides in engineered systems such as pipelines is widespread (Machuca Suarez et al., 2019). Glutaraldehyde (GLUT) for example is the most widely used commercial biocide (Kjellerup et al., 2009; Simões et al., 2011). GLUT has been an effective antimicrobial, although some reports suggest that this efficiency is reduced against multi-species biofilms (Simões et al., 2011). Although not corrosive, GLUT is not a corrosion inhibitor and is not effective at limiting attachment of bacteria (Simões et al., 2011). While mature biofilms are important targets, biocides should also control attachment of bacteria in order to prevent recovery of the population. Unlike single-function compounds such as GLUT, multi-functional compounds can inhibit corrosion as well as limit biofilm formation (Tuck et al., 2021). The primary aim of multi-functional compounds is to reduce the amount or number of compounds used in dosing protocols. If corrosion and biofilm effects can be mitigated effectively with multi-functional compounds, there are major economic advantages, time can be saved at dosing intervals and human or environmental toxicity can be reduced.

In a previous communication, a novel multi-functional organic corrosion inhibitor (OCI) compound, CTA-4OHcinn, was evaluated for its effectiveness at reducing the initial stages of biofilm formation (Tuck et al., 2021). This study demonstrated the effective reduction of the initial stages of biofilm formation (within 24 h of exposure) by three marine isolates. Although CTA-4OHcinn demonstrated promise as a corrosion inhibitor and biocide, the efficacy of this compound when applied to natural biofilms remains unknown. The present research aims to assess CTA-4OHcinn as a chemical treatment for mature, multi-species biofilms and expand the scientific understanding of this compound.

Based on a previous study, it was hypothesized that 10 mM CTA-4OHcinn would be effective at controlling multi-species biofilms (Tuck et al., 2021). To evaluate this hypothesis, biofilms were developed using an environmental consortium obtained from a West-Australian oilfield and grown on carbon steel (CS) over a 2 week period in marine-simulating conditions. These biofilms were then exposed to 10 mM CTA-4OHcinn for 4 h. Cell quantities calculated using the mean probable number (MPN) method, total adenosine triphosphate (tATP) quantification, confocal laser scanning microscopy (CLSM), post-image analysis and SEM was used to evaluate the effects of the compound on multi-species biofilms. In a novel application of the stain Cellbrite™ Fix 555, the ability of CTA-4OHcinn to cause lysis by targeting the cell membrane of a bacterial isolate was assessed.

## 2 Materials and Methods

### 2.1 Microbial Consortium

Produced water samples collected from a West-Australian oilfield were used to recover microorganisms. Aliquots of 100 ml produced water was filtered using a sterile 0.2 μm pore size membrane filter (Whatman^®^). Each filter was then placed in anaerobic SPP culture media as previously described (Salgar-Chaparro et al., 2020b) and incubated at 40°C. Subsequently, enrichment cultures were developed using media selective for sulphate producing prokaryotes (SPP), methanogenic bacteria and archaea (MET), acid producing bacteria (APB) and iron reducing bacteria (IRB). The microbial consortium for inoculation of reactors was established by pooling samples of the different microbial enrichment cultures. Enrichment samples were washed in phosphate-buffered saline (PBS, Sigma, pH 7.4) to remove wastes. Cells from each enrichment culture were then enumerated using a Neubauer haemocytometer and added in equal numbers to a final pooled inoculum. The consortium enrichment cultures were characterized, revealing *Shewanella* as the most abundant genus (52–67%). *Thermovirga* sp., *Caminicella* sp., *Pseudomonas* sp., *Paramaldivibacter* sp., *Proteiniphilum* sp. were also detected. For CLSM analysis of cell membrane disruption, a laboratory strain of *Klebsiella pneumoniae* was used as a model. Cells were cultivated in log phase for 24 h, suspended in phosphate-buffered saline (PBS, Sigma, pH 7.4) and washed twice by centrifugation at 12,000 x g for 2 min per cycle.

### 2.2 Reactor Setup and Test Conditions

**FIGURE 1:**
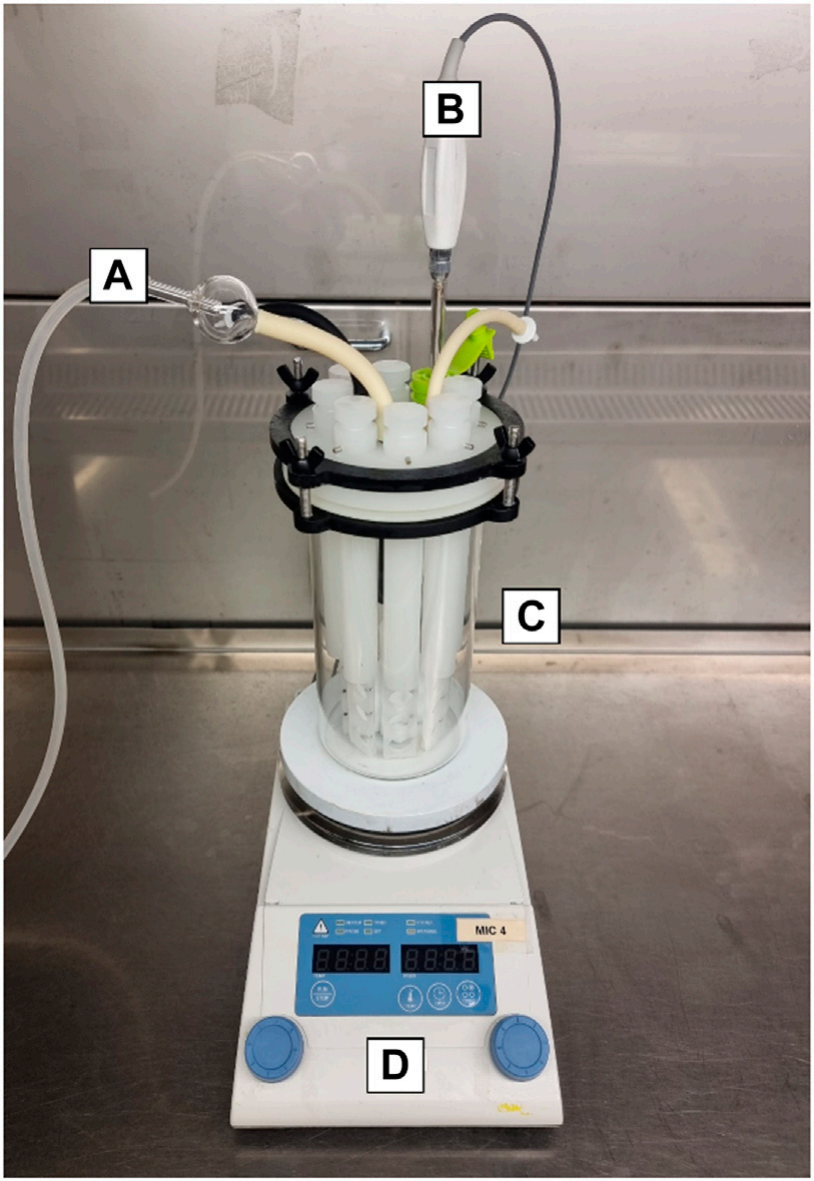
CDC CBR-90 Anaerobic Reactor design consisting of a sealed lid with gas and media inlet and outlet ports **(A)**, thermocouple probe **(B)** and glass body **(C)**, mounted on a hotplate **(D)**. Inside the reactor are coupon holding rods and a central magnet for media agitation.

A Center for Disease Control (CDC) CBR-90 Anaerobic Biofilm Reactor^®^ (Figure 1) was established in a biological safety cabinet (sterile conditions) to grow biofilms on carbon steel (AISI 1030) coupons. The coupons were placed into rods, irradiated for 10 min under UV radiation and then inserted into the reactor. The reactor gas atmosphere was established with a ratio of 20/80 CO_2_/N_2_ respectively (1 bar). Sterile test solution was pumped into the reactor after 1 h of deoxygenation with 20/80 CO_2_/N_2_ gas. Solutions and gas were sterilised by micro-filtration (0.2 μm, Whatman^®^). The reactor was placed over a stirring hot plate to maintain a temperature of 40°C with gentle agitation (50 rpm) throughout the experiment. After the temperature was established, the reactor was inoculated with the pooled microbial consortia at a concentration of 1 × 10^7^ cells/mL. To allow surface colonization, continuous flow was delayed for 36 h. After the stagnant period elapsed, continuous flow was maintained by a reservoir reactor attached to a peristaltic pump that exchanged 30% of the test solution every 24 h. These conditions ensured microbial activity was maintained throughout the experiment. Control biofilm development was assessed before CTA-4OHcinn treatment.

### 2.3 Carbon Steel Coupons

All experiments were conducted using a substrate comprised of a commercial grade of carbon steel (CS, AISI 1020). Cold finished round CS1020 bars of 12.6 mm diameter were cut into coupons 5 mm thick, before electro-coating with a protective epoxy (Powercron 6000CX, PPG Industrial coatings). Coupons were wet-ground with successively finer silicon-carbide abrasive paper in the order: 80, 120, 320, and 600 grit, to expose one side to the test solution. Each coupon had a final exposed area of 1.27 cm^2^. The ground specimens were washed with ultra-pure deionised (DI) water (18.2 MΩ.cm^2^, Milli-Q), degreased with acetone, washed with ethanol, and dried with nitrogen gas. Before applying to experiments, coupons were sterilised using ultraviolet (UV) radiation for 15 min on each side.

### 2.4 Test Solution

Produced seawater used in the present study (water injected into the process stream to improve product recovery in oil and gas operations (Yeung et al., 2011)) was collected from a West-Australian oil refinery where MIC was suspected. Produced water was supplemented with (final concentrations): 2 mM sodium thiosulphate (Na2S2O3), 6.1 mM sodium acetate (C2H3NaO2), 7.4 mM sodium formate (HCOONa), 4.7 mM sodium DL-lactate (CH3CH(OH)COONa), 4.5 mM sodium pyruvate, 5 mM glucose, 1 mM bacto™ casamino acids, 71.4 mM sodium bicarbonate (NaHCO3), 10 ml wolfe’s vitamin solution and 990 ml produced water. The final pH of the test solution was 7.2–7.5.

### 2.5 Biocide Treatment

Biocide treatment was applied after 2 weeks of biofilm growth. After 2 weeks, coupons yielded mature, complex biofilm structures with visible EPS. Before the biocide injection, the CDC reactor was drained and rinsed with a fresh deoxygenated test solution to eliminate waste products or dead cells. Control samples were extracted to perform microbiological and microscopic analyses before the biocide injection. The reactor was again drained and finally replenished with a fresh deoxygenated test solution containing 10 mM of CTA-4OHcinn. After 4 h of exposure, the remaining samples were collected to assess the effect of the biocide on the biofilm viability and structure.

### 2.6 Microbiological Analyses

Coupons were rinsed with sterile PBS (Sigma, pH 7.4) and immersed in sterile tubes with 10 ml of anaerobic PBS solution before and after biocide injection. Seven sonication cycles (10 s ON followed by 20 s OFF) were performed to detach microorganisms from the coupons. Samples were immersed in ice during OFF cycles to prevent heat damage. The remaining suspension was then used to conduct the microbial analysis (MPN and ATP) described below.

#### 2.6.1 Mean Probable Number (MPN) Cell Quantity Estimations

MPNs were determined as described in the Microbiological Examination Methods of Food and Water: A Laboratory Manual (da Silva et al., 2019). Briefly, a 1 ml cell suspension from each coupon was inoculated into 9 ml of anaerobic SPP culture medium serially diluted 10-fold in triplicate for the MPN estimation. Serial dilution vials were incubated at 40°C for 28 days, and positive growth was determined by visual inspection of changes in media turbidity and the colour of the culture media, according to a standard protocol outlined elsewhere (da Silva et al., 2019). Positive vials were confirmed by phase-contrast microscopy using a Nikon Eclipse Ci-L. The microbial concentration was determined using the three-tube standard table for MPN quantification assays outlined in the standard (da Silva et al., 2019).

#### 2.6.2 Adenosine Triphosphate (ATP) Assays

Total biofilm ATP was quantified on coupons before and after exposure to CTA-4OHcinn. Experiments were conducted using the Quench-Gone Organic Modified test kit (QGO–M™; Luminultra Technologies Ltd.) according to the manufacturer’s instructions, with the exception of a sonication step as described above during the 5 min Lumilyse™ incubation. ATP measurements were obtained using the PhotonMaster™ Luminometer (Luminultra Technologies Ltd.), and ATP content was calculated from the measured luminescence by comparison with a standard.

#### 2.6.3 Statistical Analysis

A one-way ANOVA statistical analysis was conducted on MPN and ATP triplicate data points using PAST (V4.03) (Hammer et al., 2001) to determine if differences between MPN and ATP data before and after treatment were significantly different. A one-way ANOVA statistical analysis on CLSM micrographs was also conducted to verify that fluorescent signal obtained from CTA-4OHcinn treated samples was significantly greater than untreated samples. Results were considered significant upon returning a *p*-value ≤ 0.05.

### 2.7 Scanning Electron Microscopy

Coupons from before and after biocide exposure were removed from reactor rods and lightly rinsed in PBS at 30°C (Sigma, pH 7.4) before fixation in 2.5% glutaraldehyde for 22 h at 4°C. Fixed coupons were placed under nitrogen gas overnight to completely dry before sputter coating with 9 nm of platinum. This procedure was developed to minimise the effects of charging caused by non-conductive biofilm-covered surfaces. Microscopy was conducted using a Neon field emission scanning electron microscope equipped with a secondary electron detector. An emission voltage of 5 kV, aperture of 30 µm and working distance of 6 mm (adjusted to 5.8 and 5.9 mm during focusing) was used to image surfaces before and after exposure to CTA-4OHcinn.

### 2.8 Confocal Laser Scanning Microscopy

CLSM was conducted on a Nikon A1+ confocal microscope equipped with the latest version of Nikon Elements software and a 20 x dry objective lens. Coupons from before and after biocide exposure were stained according to the manufacturer’s instructions. Briefly, coupons were immersed for 10 min in the Syto9™ and propidium iodide (PI) mixture (Invitrogen™ Filmtracer™ LIVE/DEAD™ Biofilm Viability Kit) and lightly rinsed in PBS (Sigma, pH 7.4). Micrographs were captured sequentially using 489.3 and 561 nm lasers and 500–550 nm and 570–620 nm emission filters respectively. Spectral bleed-through was reduced by acquiring z-stack images in separate tracks for emission and excitation paths. Two random locations were sampled from each coupon using the Nyquist function for semi-quantitative analysis. The low magnification (20 x) was selected to capture a larger sample size of 182 μm^2^. A third micrograph was captured to represent the coupon surface through 3D reconstruction (20 x full field of view, measuring 600 × 600 µm). All micrographs were captured using the same microscope and software settings for uniformity.

To visualise the effect of CTA-4OHcinn on bacterial membranes, Cellbrite™ Fix 555 (Biotium) was used in a 10 x concentration to stain *K. pneumonia* cells. Cells from the same culture were stained (controls) or combined with 10 mM CTA-4OHcinn and stained, before transferring 10 µL to a glass slide. Since Cellbrite™ Fix 555 stains do not require washing, microscopy was performed after 1 h of exposure to the stain or stain and CTA-4OHcinn mixture. A glass cover slip of 0.17 mm thickness was placed over the droplet and sealed using clear epoxy resin. Microscopy was conducted using a transmission detector and 561 nm laser with a 595/50 emission filter. A 100 x oil immersion objective was used to visualise individual cells, maintaining microscope settings between control and experimental samples. Triplicate micrographs were then captured across random areas of untreated control and treated samples for post-image analysis. The micrographs are also presented with combined and separate signals to depict the cell morphology and membrane damage (indicated by fluorescence intensity and distribution) under control and treated scenarios.

#### 2.8.1 Post-image Analysis

Replicate Nyquist micrographs from each surface were captured randomly and used to estimate biofilm parameters including % live and % dead cells, relative biomass (expressed as mean signal intensity) and compactness. Z-stacks were converted and loaded into the latest version of IMARIS (Bitplane) software (9.7.2), generating a series of statistics used to calculate the following parameters: compactness (a measure of signal density per area sampled), % live cells on each surface, % dead cells on each surface and relative mean signal intensity for each channel on each surface (red and green, or live and dead).

Micrographs from Cellbrite Fix 555 stained *K. pneumoniae* were also processed using IMARIS (Bitplane) software to quantify signal from membrane compromised cells. A comparison of triplicate micrographs captured after 1 h was made by comparing the untreated control against a sample treated with 10 mM CTA-4OHcinn. Membrane compromised cells were discriminated using background subtraction, eliminating signal from live cells leaving only signal from membrane compromised cells for quantification. Triplicate micrographs from untreated control and treated samples were compared using the same analysis method and the same threshold values.

## 3 Results and Discussion

Biofilms in marine environments are responsible for biofouling and MIC, and are managed in engineered systems such as pipelines using mechanical cleaning and biocides (Okoro, 2015). Some of the most effective and widely used biocides in engineered systems and healthcare are associated with toxic effects to people (Smith and Wang, 2006) and the environment (Leung, 2001; Emmanuel et al., 2005; Sano et al., 2005), and are usually dosed in parallel with other compounds such as corrosion inhibitors (Zhong et al., 2020). The choice of corrosion inhibitor in a given system is dependent on a number of other factors; including but not limited to flow rate of pipelines, produced water composition, H_2_S and CO_2_ content and suspected corrosion mechanisms (Mesquita and Marchebois, 2020). Thus, a variety of organic and inorganic compounds have been developed to address corrosion concerns in oil and gas operations.

In the context of expanding limitations imposed by environmental guidelines, “greener” options are gaining increasing traction in the scientific literature (Chugh et al., 2020). Organic plant extracts for example can be affordable and effective at low concentrations, i.e. ginger extract at 20 ppm can achieve corrosion inhibition efficiency of ∼80% on CS (Chugh et al., 2020). Similarly multifunctional organic compounds (such as those based on QACs) are a promising new strategy to combat MIC and other corrosion types with reduced cost, toxicity and environmental impact (Zhong et al., 2020). Along with reduced compound toxicity, corrosion inhibitors with biocidal properties such as CTA-4OHcinn eliminate the need for these functions to be addressed by individual compounds, thus reducing exposure to personnel and dosing costs (Zhong et al., 2020).

Previous research evaluated the corrosion inhibition efficacy of CTA-4OHcinn on CS (AISI 1030) applied at 5, 7.5, and 10 mM, finding 95, 95, and 96% corrosion inhibition efficiency respectively after 30 min (Ghorbani et al., 2020). The results of corrosion inhibition efficiency experiments indicate that CTA-4OHcinn shows great promise as a corrosion inhibitor. Based on the concentrations applied in this study, 10 mM was applied in a subsequent study to evaluate the effect of CTA-4OHcinn on early biofilm formation (Tuck et al., 2021). This earlier study evaluated the attachment and colonization of three isolates exposed to the compound using CDC reactors. The results indicated that CTA-4OHcinn reduced bacterial viability by between 96 and 100% on wet-ground and oxidised CS substrates (Tuck et al., 2021). Although these results support biocidal efficacy of CTA-4OHcinn, a greater understanding of the susceptibility of mature biofilms is required before practical application of CTA-4OHcinn can be considered.

Natural biofilms are characterized by species diversity, giving rise to complex and poorly understood synergistic and competitive interactions (Guillonneau et al., 2018). Higher abundance of species in natural biofilms is also associated with greater tolerance to biocidal compounds, although limited work explores the mechanisms governing this tolerance (Sanchez-Vizuete et al., 2015). The present study investigates the efficacy of CTA-4OHcinn in the context of developed biofilms grown using multi-species oilfield samples to better reflect recalcitrant environmental populations. *Shewanella* sp, *Paramaledivibacter* sp. and *Pseudomonas* sp., previously identified marine taxa, represented the dominant species of the consortium. Although little is known about *Paramaledivibacter* sp., the *Shewanella* and *Pseudomonas* genus are frequently associated with MIC of steels in marine environments (De Windt et al., 2003; Yuan et al., 2007; Machuca Suarez et al., 2016; Salgar-Chaparro et al., 2020a; Wurzler et al., 2020).

**FIGURE 2:**
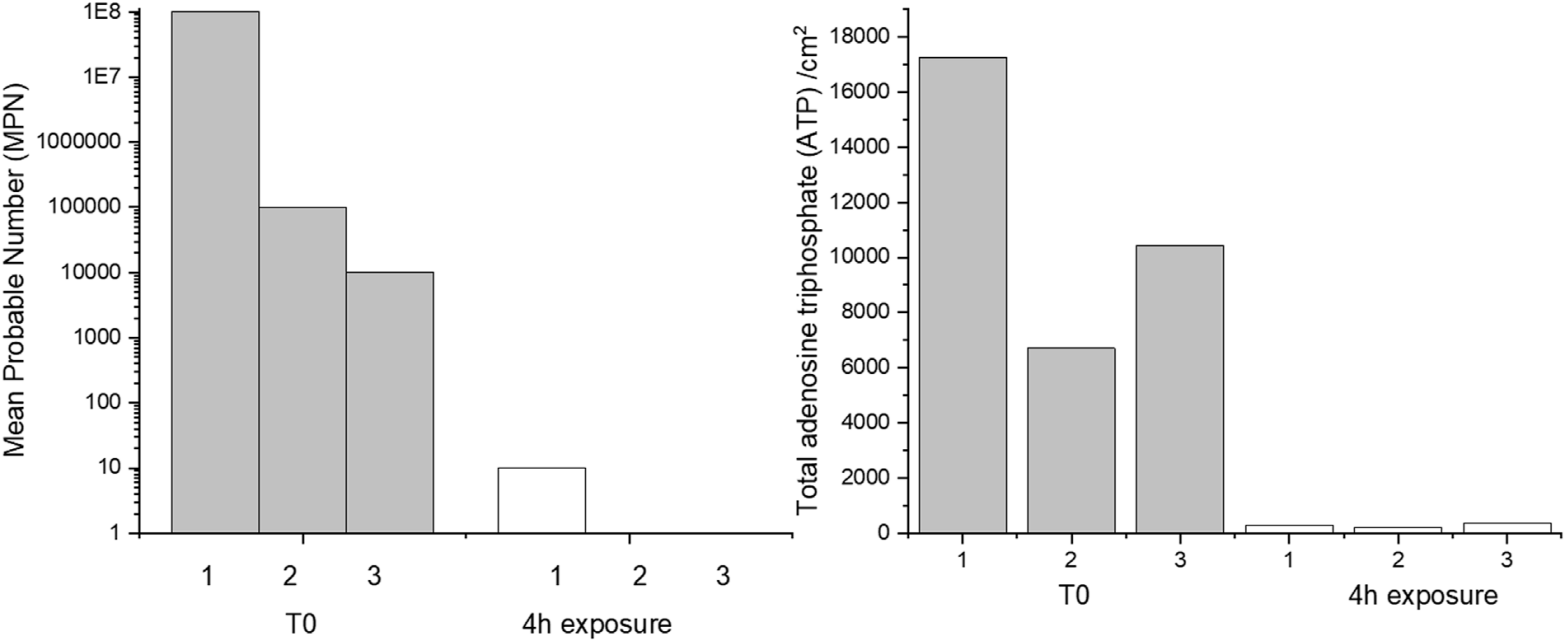
Triplicate mean probable number (MPN, left) and tATP measurements (right) taken from before (T0) and after 4 h of exposure to CTA-4OHcinn.

The effect of 4 h of exposure to CTA-4OHcinn on cell viability and biofilm metabolic energy of 2-week-old biofilms were evaluated using mean probable numbers (MPN) and total adenosine triphosphate (tATP) (Figure 2). Results from both assays suggest that at T_0_ (before dosing with CTA-4OHcinn), biofilms were healthy. A statistically significant reduction in both viable cells and tATP was observed after exposure to CTA-4OHcinn. Regardless of the number of cells initially detected in healthy biofilms, MPN and tATP measurements indicated that most, but not all cells were killed and removed by CTA-4OHcinn.

**FIGURE 3:**
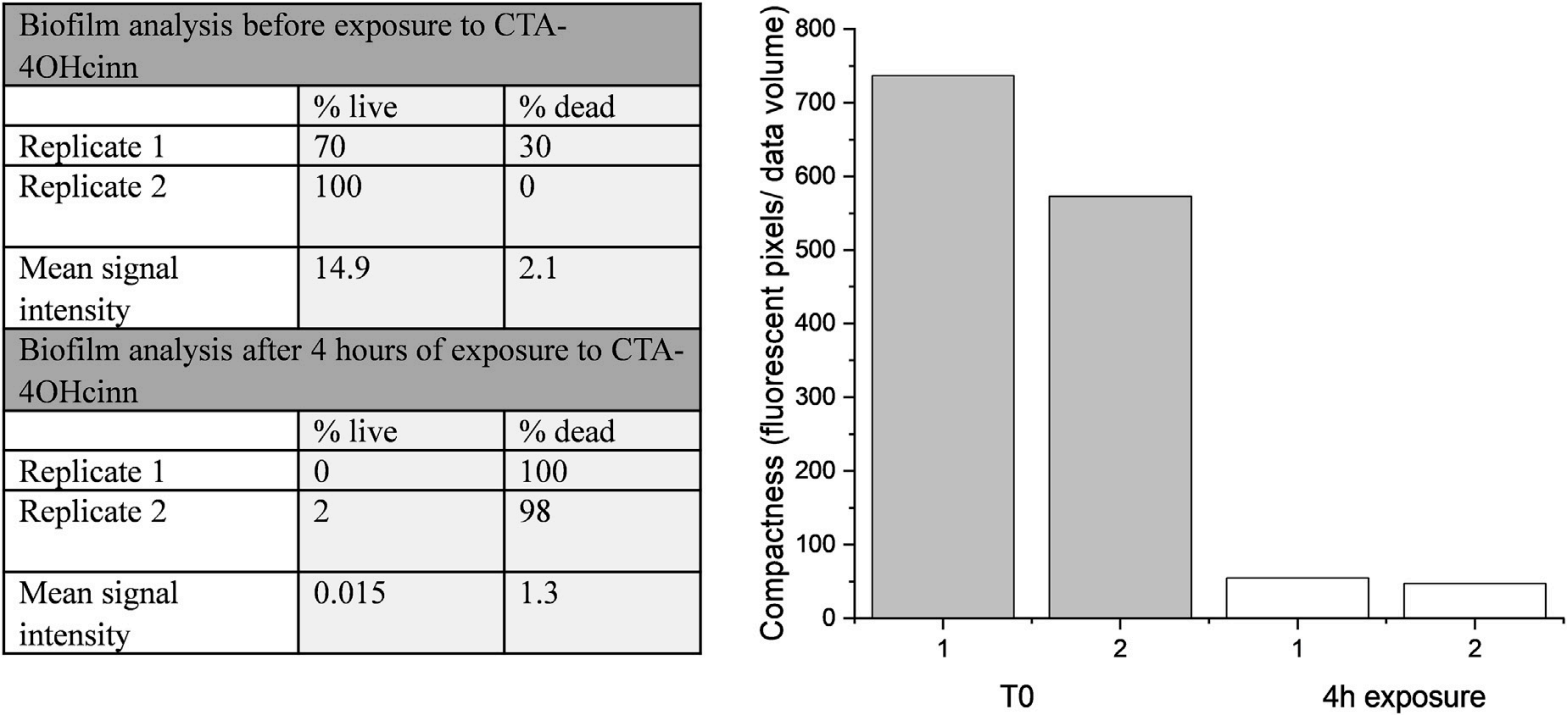
IMARIS (Bitplane) analysis of replicate Nyquist CLSM micrographs as % live cells and % dead cells, and mean signal intensity from green and red channels (left). Fluorescent pixels (pixels that returned a green or red signal) divided by the total volume of data provides an estimation of biofilm compactness (right). This data was constructed from micrograph compactness values before (T_0_) and after 4 h of exposure to CTA-4OHcinn.

Confocal laser scanning microscopy (CLSM) and post-image analysis indicates that almost all biofilm was killed and removed by the biocide (Figure 3). CLSM dyes bind to DNA and can distinguish live (Syto9™- green signal) and dead or damaged (propidium iodide-red signal) cells through membrane permeability. Once bound to the target, fluorescent signal is greatly enhanced. Post-image analysis can provide a 3D reconstruction of the surface based on the fluorescent signal returned. The spatial distribution and quantity of live and dead/damaged cells can then be visualized or represented numerically through quantitation of biofilm parameters. In the present study Bitplane (IMARIS) software was selected for this purpose. According to this analysis, biofilm compactness was reduced 10 fold in the presence of CTA-4OHcinn and biofilm composition changed from a mean of 85–1% live cells after the addition of CTA-4OHcinn. The greatly reduced mean combined signal intensity from both channels after exposure indicates that CTA-4OHcinn may work as a surfactant. Combined intensity values were reduced 12 fold (Figure 3), implying a significant reduction in surface adhered biofilm. This data supports MPN and ATP calculations, indicating that the biocide was effective at reducing viability as well as biomass of the biofilm.

**FIGURE 4:**
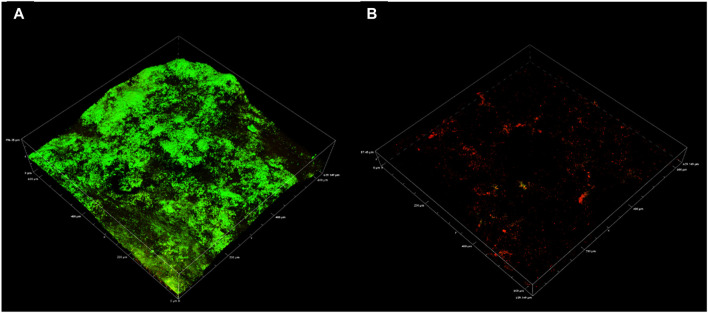
Representative confocal micrographs captured using a 20 x objective depicting biofilms on CS. Micrographs were captured on coupons with 2 week old biofilms **(A)** and on 2 week old biofilms exposed to CTA-4OHcinn for 4 h **(B)**. Green indicates live cells and red indicates dead or damaged cells.

CLSM 3D reconstructions of the biofilm were also generated to compare biofilm architecture and distribution of live and dead cells (Figure 4). Biofilm CLSM reconstructions show a strong green signal (live cells) in untreated biofilms (Figure 4), compared to treated biofilms where almost no green signal was observed (Figure 4). Figure 4B also demonstrates low combined signal from both channels with little biofilm architecture remaining after treatment. For lethal biocides, lysis is expected to change the cell signal to red, leaving the biofilm architecture relatively undamaged. This result was achieved in a confocal study where ethanol treated and untreated multi-species biofilms were compared. Ethanol treatment resulted in cell lysis, indicated by a red signal, while untreated biofilms remained mostly viable as indicated by a green signal (Samarian et al., 2014). The results from that study demonstrate that when simply killed but not removed, propidium iodide fluorescence reveals the biofilm architecture in red. CLSM results in the present communication indicate that CTA-4OHcinn functions as a biocide as supported by viability assays, and also as a dispersal agent as indicated by significantly reduced biofilm architecture at the interface.

**FIGURE 5:**
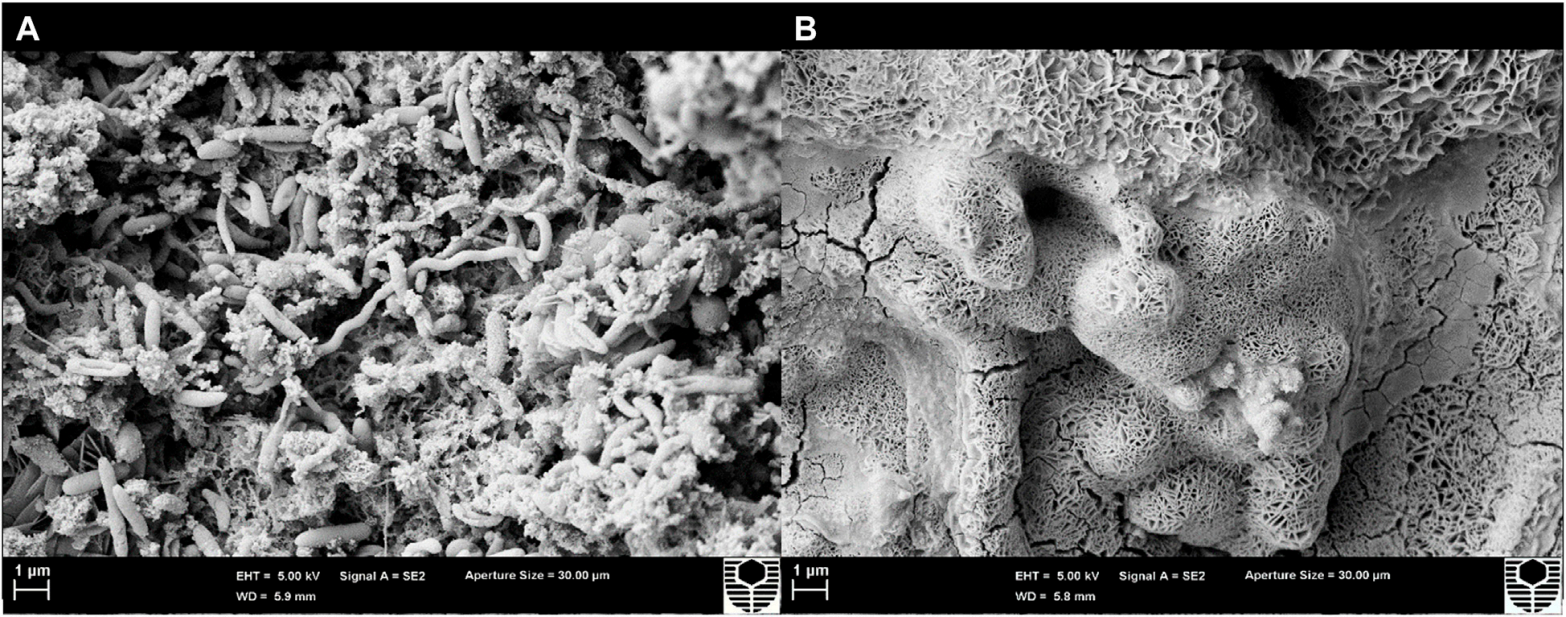
Scanning electron micrographs of a coupon surface captured before **(A)** and after 4 h exposure to CTA-4OHcinn **(B)**. Surface A shows structures resembling bacterial cells not observed on surface B.

Scanning electron microscopy (SEM) provided a visual comparison between the biofilm at T_0_ and after treatment with CTA-4OHcinn. Figure 5A shows a complex, multi-species biofilm with structures resembling various morphologies on the surface at T_0_. The absence of these structures after exposure (B) indicates successful lysis and removal of cells.

The present study found CTA-4OHcinn to be effective at reducing the viability and architecture of mature, multi-species marine biofilms under reactor conditions. While the results are in agreement with a previous study (Tuck et al., 2021), comparisons of CTA-4OHcinn performance, especially compared to results from established biocidal compounds (such as glutaraldehyde or tetrakis (hydroxymethyl)phosphonium sulphate, are still required. CTA-4OHcinn functions simultaneously as a corrosion inhibitor and biocide, and thus scientifically valid comparisons are difficult to achieve. Future research aims to compare CTA-4OHcinn with commercial biocides in consideration of multiple functions.

Environmental toxicity is also critical to consider when introducing novel biocides for application in marine environments. QACs demonstrate low LD_50_ values when compared against commercial biocides such as glutaraldehyde (Seter et al., 2013). In the present study, CTA-4OHcinn is considered environmentally sensible as a QAC, with a recent study confirming low LD_50_ values and adsorption properties against human keratinocyte (skin) and duodenum (intestinal) cell lines (Catubig et al., 2021). The communication found the CTA-4OHcinn toxicity profile to be comparable to cetrimonium bromide; a safe and widely applied additive in cosmetic products and disinfectants (Catubig et al., 2021). Additionally, the multifunctional nature of CTA-4OHcinn eliminates the requirement to treat biofilm formation and corrosion with separate compounds.

As a novel compound, the biocidal mechanism for CTA-4OHcinn remains unknown. Since the cetrimonium cation is associated with biocidal activity through cell membrane disruption, it was proposed that CTA-4OHcinn will exert similar effects (Ittoop et al., 2015). To determine if the membrane-disruptive function of the cetrimonium cation was maintained in CTA-4OHcinn, healthy cultures of *K. pneumoniae* were treated with CTA-4OHcinn and compared to untreated controls by staining with Cellbrite™ Fix 555.

**FIGURE 6:**
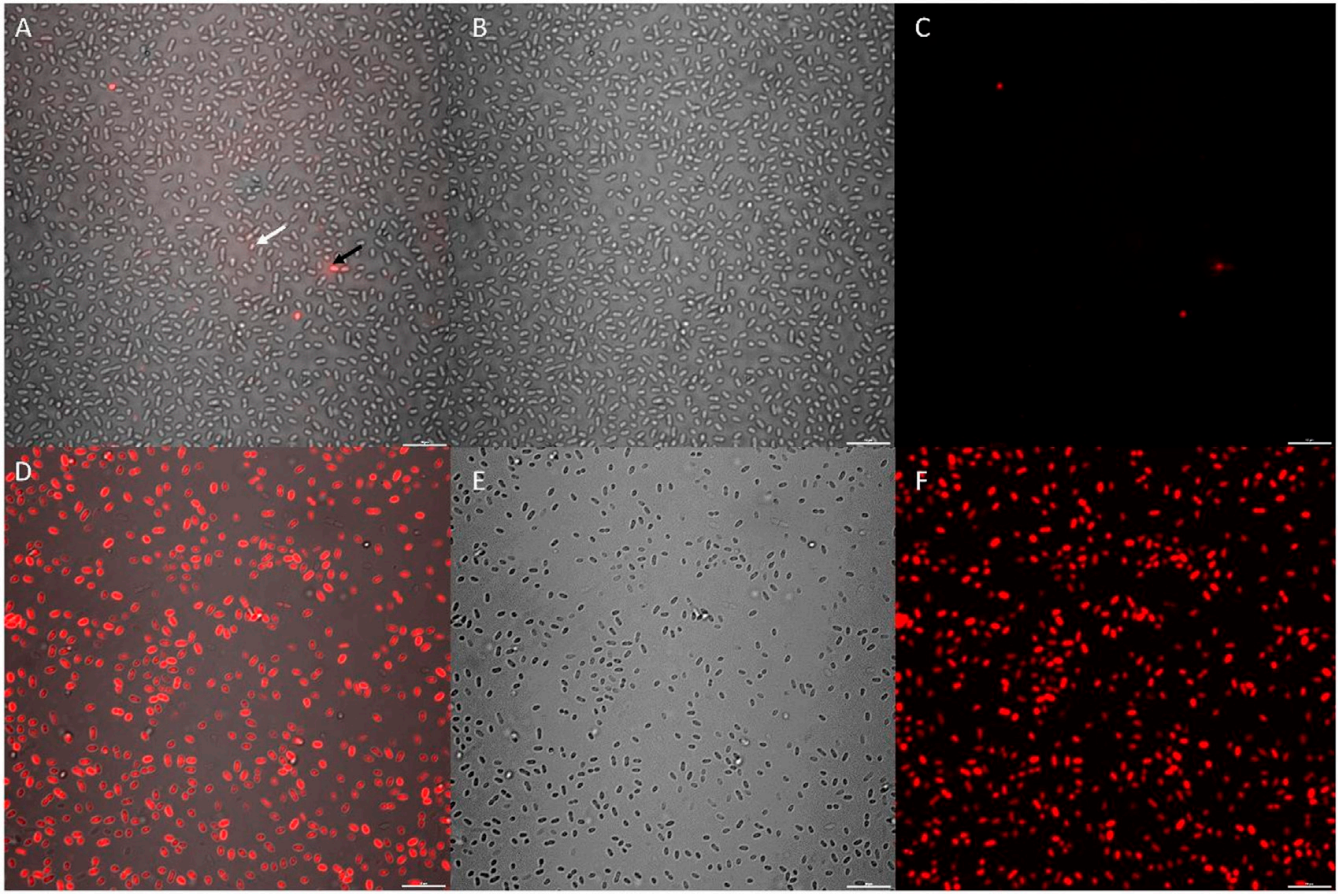
CLSM micrographs depicting *K. pneumoniae* cells stained with a 10 x concentration of Cellbrite Fix 555; where **(A–C)** = untreated control sample and **(D–F)** = CTA-4OHcinn treated sample. **(A,D)** compare combined channels, **(B,E)** compare transmission detector channels and **(C,F)** compare fluorescent signal only. The white arrow indicates a live cell with low Cellbrite Fix 555 signal (membrane fluorescence) and the black arrow indicates a membrane compromised cell emitting greater signal.

High magnification CLSM post-image analysis comparisons required a small, dense and uniform cell sample. Variations in multi-species consortium samples can skew downstream analysis results, and thus a single species culture was preferential. *K. pneumoniae* was selected for these experiments based on rapid reproduction in enriched seawater and large, easily visible cell morphology under 100 x magnification. Additionally, the isolate endured washing stages with minimal damage to cell membranes, as evidenced by controls (Figure 6A).

Cellbrite™ Fix 555 was selected as a general membrane stain to visualize damage caused by CTA-4OHcinn under CLSM. Cellbrite™ Fix 555 is an amine-reactive fluorescent dye that emits a low red signal when in contact with membrane proteins. Due to covalent binding of the stain to amines, fluorescent signal is greatly enhanced in cells with membrane damage since intracellular proteins contain abundant binding sites. Therefore, Cellbrite™ Fix stains represent an ideal tool to visualize cell membrane disruption in bacteria. In post-image analysis, fluorescent signal was quantified only from intracellular fluorescence to eliminate live cells from this analysis (low signal intensities not corresponding to cell damage).

*K. pneumoniae* cells were treated with CTA-4OHcinn for 1 hour and compared to the same cultures without treatment (Figure 6). Cell samples were denser in untreated controls compared to cells treated with CTA-4OHcinn (Figures 6B,E). Since experimental replicates cannot control for exact cell numbers, micrographs for post-image analysis were captured favoring more cells in the field of view for untreated samples. Bias introduced by sample size was eliminated in this way to ensure significance was maintained.

Lastly, the mean sum of fluorescent signal returned from triplicate control micrographs was 2.4E7, compared to 2.5E8 returned from treated samples. Thus, a 10 fold average increase in fluorescent signal intensity in CTA-4OHcinn treated samples was observed (Figures 6A,C,D,F). The comparison between treated and untreated samples by post-image analysis revealed a statistically significant difference (p = ≤0.002), confirming that CTA-4OHcinn acts on the cell membrane to cause cell lysis.

This study provides the first evidence that application of a novel organic corrosion inhibitor, CTA-4OHcinn, functions as a biocide and is effective against multi-species biofilms developed on CS. Further, the investigation suggests that CTA-4OHcinn biocidal mechanism is membrane disruption. In similar QACs, the positive charge exerts an electrostatic interaction with negatively charged bacterial membranes (Seter et al., 2013). After initial adsorption, the quaternary ammonium cation moiety is able to diffuse through the cell wall (Seter et al., 2013). The downstream effects of the cetrimonium cation permeation are phospholipid bilayer destabilization resulting in cell death (Schachter, 2013; Iñiguez-Moreno et al., 2018). Additionally, the cation could result in cell lysis through its quenching action against ATP synthase, ultimately resulting in energy deprivation (Schachter, 2013). It is also hypothesized that the *trans-*4OHcinn anion is capable of contributing to the antimicrobial activity of the cation, although the interactions involved, and synergism between the cation and anion in the present study remains unknown and is a topic under further investigation.

## 4 Conclusion

Demand for environmentally friendly and cost-effective biocides has never been higher. By developing corrosion inhibitors with biocidal properties, the number of chemicals used at dosing intervals can be reduced along with costs and environmental impact. For multi-functional compounds to be attractive for use in industrial systems such as pipelines, they must be effective at inhibiting both mature biofilms and bacterial attachment. In a previous study, a novel, multi-functional organic corrosion inhibitor designated CTA-4OHcinn was found to have anti-corrosive properties and biocidal properties against bacterial attachment and early biofilm formation. In light of these studies, the present research aimed to understand the effect of this compound on more mature, multi-species biofilms developed using a West-Australian oilfield consortium. The application of 10 mM CTA-4OHcinn was recently found to be optimal for corrosion inhibition. In this study, 4 h of treatment with 10 mM CTA-4OHcinn significantly reduced biofilms as demonstrated by reduced cell numbers and total adenosine triphosphate (tATP) values. Biofilm dispersal qualities in CTA-4OHcinn were also observed. Confocal micrographs and post-image analysis indicate that biofilm compactness, live cell quantity and cell viability was significantly reduced after biocide application; with few live or dead cells remaining on the treated surface. Under scanning electron microscopy, structures resembling a complex, multi-species biofilm were present in healthy control biofilms but were not present in the same biofilms after CTA-4OHcinn treatment. Lastly, CTA-4OHcinn was found to act on the cell membrane of bacterial cells. This study introduces CTA-4OHcinn as an effective biocide for use against multi-species biofilms. The compound is among the first of its kind to be found effective at simultaneously mitigating corrosion as well as limiting early and mature biofilm formation.

## Data Availability

The original contributions presented in the study are included in the article/Supplementary Material, further inquiries can be directed to the corresponding author.
